# Modularized microscope based on parallel phase-shifting digital holography for imaging of living biospecimens

**DOI:** 10.1117/1.JBO.25.12.123706

**Published:** 2020-12-04

**Authors:** Junya Inamoto, Takahito Fukuda, Tomoyoshi Inoue, Kazuki Shimizu, Kenzo Nishio, Peng Xia, Osamu Matoba, Yasuhiro Awatsuji

**Affiliations:** a Kyoto Institute of Technology, Graduate School of Science and Technology, Kyoto, Japan; b Japan Society for the Promotion of Science, Tokyo, Japan; c National Institute of Advanced Industrial Science and Technology, National Metrology Institute of Japan, Tsukuba, Japan; d Kobe University, Graduate School of System Informatics, Department of Systems Science, Kobe, Japan

**Keywords:** microscope, holographic microscopy, digital holography, digital holographic microscopy, three-dimensional trajectory, three-dimensional tracking

## Abstract

**Significance:** Parallel phase-shifting digital holographic microscope (PPSDHM) is powerful for three-dimensional (3D) measurements of dynamic specimens. However, the PPSDHM reported previously was directly fixed on the optical bench and imposed difficulties case, thus it is required to modify the specification of the microscope or transport the microscope to another location.

**Aim:** We present a modularized PPSDHM. We construct the proposed PPSDHM and demonstrate the 3D measurement capability of the PPSDHM.

**Approach:** The PPSDHM was designed as an inverted microscope to record transparent objects and modularized by integrating the optical elements of the PPSDHM on an optical breadboard. To demonstrate the effectiveness of the PPSDHM, we recorded a 3D motion-picture of moving *Volvoxes* at 1000  frames/s and carried out 3D tracking of the *Volvoxes*.

**Results:** The PPSDHM was practically realized and 3D images of objects were successfully reconstructed from holograms recorded with a single-shot exposure. The 3D trajectories of *Volvoxes* were obtained from the reconstructed images.

**Conclusions:** We established a modularized PPSDHM that is capable of 3D image acquisition by integrating the optical elements of the PPSDHM on an optical breadboard. The recording capability of 3D motion-pictures of dynamic specimens was experimentally demonstrated by the PPSDHM.

## 1 Introduction

**Fig. 1:**
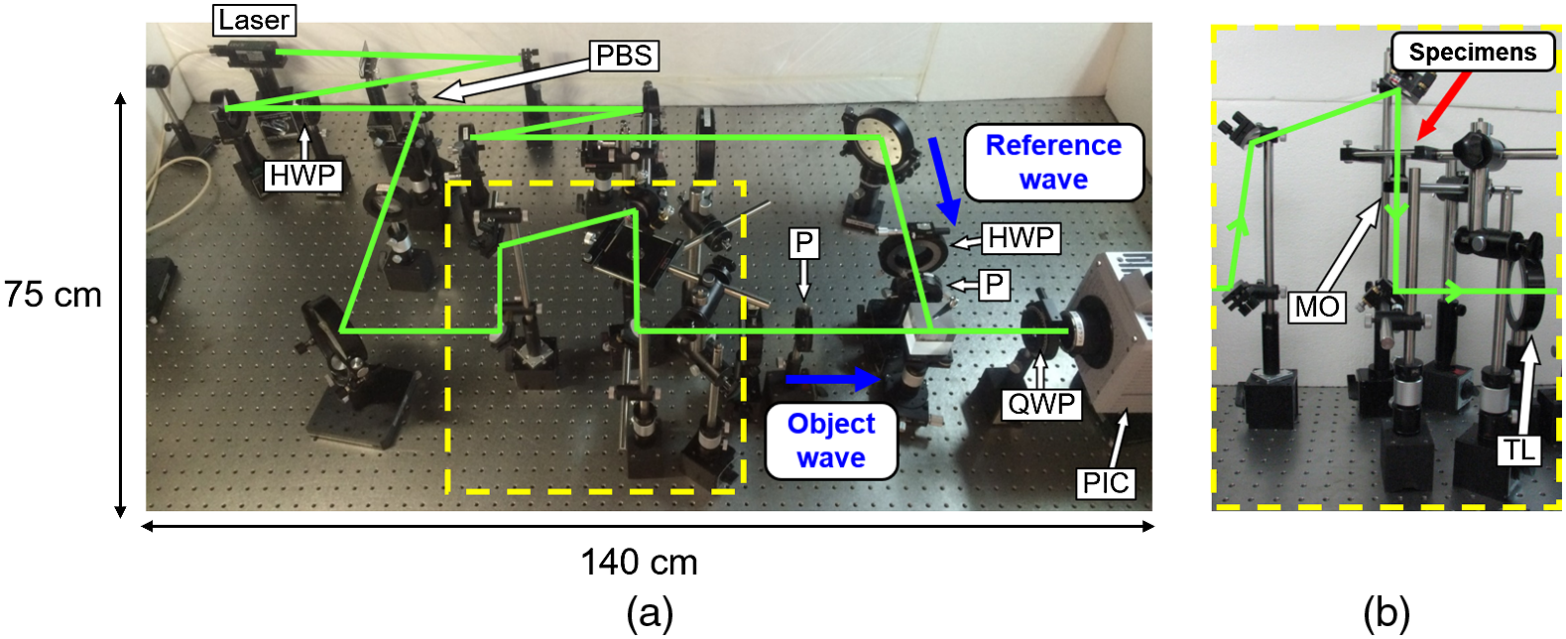
Photographs of the optical system of the previous PPSDHM: (a) top view of the whole optical system and (b) side view of the magnification optical system shown in the yellow dashed line square in (a). PBS, polarizing beam splitter; HWP, half-wave plate; QWP, quarter-wave plate; PIC, polarization-imaging camera; P, polarizer; MO, microscope objective. The green lines show the optical paths.

Three-dimensional (3D) measurements of the shape, behavior, or motion of living cells and microorganisms are useful in biology, for example, in understanding the effect of the surrounding extracellular matrix on the cell and in the classification of cells or microorganisms. In recent years, 3D measurements of biological specimen have been reported, which includes the 3D imaging of cells^1^^–^^5^ and the 3D tracking of microorganisms.^6^^–^^8^ Microscopes such as a confocal microscope,^9^^,^^10^ multiphoton microscope,^11^^,^^12^ and light sheet microscope^13^^,^^14^ are capable of 3D measurements in a micro-area. However, it is difficult for these microscopes to measure the change in the 3D shape of specimens or the 3D behavior of dynamic specimens due to the requirement of mechanical scanning of a light beam or moving the specimen stage. One of the microscopes that realizes 3D measurements without mechanical scanning is the light field microscope.^15^^,^^16^ A light field microscope is a great tool for imaging weakly scattering or fluorescent specimens with good light efficiency and fast imaging speed. However, there is a trade-off between the resolution and the depth of field of a light field microscope. In other words, the higher the resolution is in a light field microscope, the shallower the depth of field is. As a result, the ability to refocus in a light field microscope is decreased. Thus, a light field microscope is incapable of carrying out 3D measurements with the high resolution and large depth of field at the same time. By contrast, a digital holographic microscope (DHM)^17^^–^^20^ is a promising tool for 3D measurements of dynamic specimens with high resolution and large depth of field. A DHM is based on digital holography.^21^^–^^24^ Digital holography is a technique of digitally recording the interference fringes due to an object and referencing it as a hologram using an image sensor and then reconstructing the complex amplitude distribution of the object on a computer. The interference fringe is generated by the superposition of light waves containing the information on the object (object wave) and the reference wave. This method is capable of digital refocus; the technique can arbitrarily reconstruct any depth plane of the object after the recording of the object because the complex amplitude distribution of the object is numerically obtained by the technique. The larger the offset angle between the object wave and the reference wave is, the finer the interference fringe is. An image sensor cannot record a fine interference fringe when the offset angle is large. As a result, a DHM can neither deal with a large object nor provide a large field of view. Consequently, an in-line DHM that has zero offset angle is often employed. However, the image obtained by an in-line DHM is degraded in general due to the overlap of the zeroth-order diffraction wave and conjugate image on the image of the object. To solve this problem, a phase-shifting digital holographic microscope (PSDHM)^25^^,^^26^ was proposed, which is based on phase-shifting digital holography (PSDH).^27^^–^^29^ Although a PSDHM can reconstruct the image on which the undesired images are removed, it requires multiple holograms to be sequentially recorded using the reference waves with different phase retardations. Therefore, it is difficult for a PSDHM to record dynamic objects. To achieve an accurate 3D motion picture of the shape, the behavior, or the motion of dynamic specimens, a parallel phase-shifting digital holographic microscope (PPSDHM)^30^^–^^32^ was proposed. PPSDHM is a microscope based on parallel phase-shifting digital holography (PPSDH)^33^^–^^36^ and can reconstruct the image of an object where the undesired images are removed. By applying the technique of the space-division multiplexing of holograms, PPSDH records the multiple holograms required for PSDH on a single hologram with a single-shot exposure. We previously reported the 3D motion-picture imaging of minute specimens moving three-dimensionally^31^^,^^32^ using a PPSDHM. However, the optical system of the previous PPSDHM had two problems because all of the optical elements of the PPSDHM were directly fixed on the optical bench as shown in Fig. 1. First, in the case where we need to change the specification of the microscope such as the magnification, it is necessary to redesign not only the corresponding microscope but also the entire system. Second, it was difficult to transport the microscope to another location. In this paper, we report a modularized PPSDHM to solve the above problems. To demonstrate the effectiveness of the modularized PPSDHM for 3D measurements of biospecimens, we carried out the 3D tracking of *Volvox* moving in water.

## 2 Parallel Phase-Shifting Digital Holographic Microscope

**Fig. 2:**
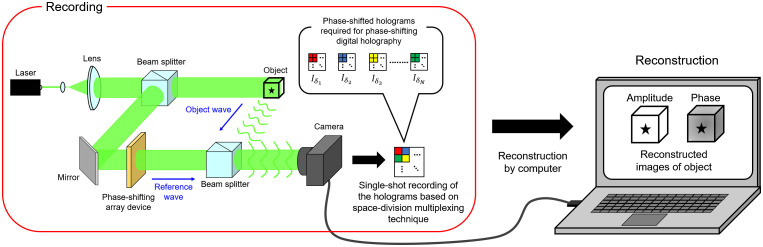
Schematic of the principle of parallel phase-shifting digital holography.

Figure 2 shows a schematic of the principle of PPSDH. PPSDH simultaneously records the multiple holograms required for PSDH in a single hologram using a space-division multiplexing technique of holograms. The space-division multiplexing is implemented by the phase-shifting array device. The multiple holograms required for PSDH are generated from the recorded hologram by the pixel extraction and interpolation of the hologram. The complex amplitude distribution of the object wave on the image sensor is computed from the multiple holograms by employing the numerical processing used in the phase-shifting interferometry. The complex amplitude distribution of the object wave at an arbitrary depth position is obtained by applying the diffraction integral (which is a mathematical operation representing the light propagation to the complex amplitude) to the complex amplitude of the image sensor plane. Because the undesired images are removed from the obtained complex amplitude, PPSDH can provide an accurate image of the object from the single hologram recorded with a single exposure. Therefore, PPSDH is applicable to a moving object. Hence, a PPSDHM is suitable for 3D measurements of living biospecimens. In particular, a PPSDHM using a high-speed camera is powerful for 3D measurements of biospecimens dynamically moving in a micro-area. This is because the speed of the enlarged image of the object moving in a micro-area is faster than the actual speed of the object.

In general, the higher magnification of the microscope is, the shallower the depth of field of the microscope is. A usual optical microscope requires mechanical scanning of a light beam or moving a specimen stage for 3D measurements because the depth of field is shallow. By contrast, a DHM can carry out 3D measurements without mechanical scanning because the depth of field of a DHM is deep even in the case of high magnification. Of course, a PPSDHM can achieve 3D measurements without mechanical scanning.

## 3 Design and Construct of a Modularized Parallel Phase-Shifting Digital Holographic Microscope

### 3.1 Design

We designed a modularized PPSDHM along the following lines.

(1) Easy interchange of the microscope where all the optical elements are integrated on an optical breadboard itself.
(2) Easy transportation to another location.
(3) Easy recording of transparent and living biospecimens.

To achieve points (1) and (2), we construct the optical system in a small space on an optical bench. For this purpose, the optical elements of the microscope, except for a camera, were integrated on an optical breadboard. The reason why the camera was arranged outside the breadboard will be described later. To achieve point (3), we adopted an inverted microscope as the configuration of the microscope. We chose an inverted microscope because this configuration was suited for the purpose of 3D observation of biospecimens better than an upright microscope.

**Fig. 3:**
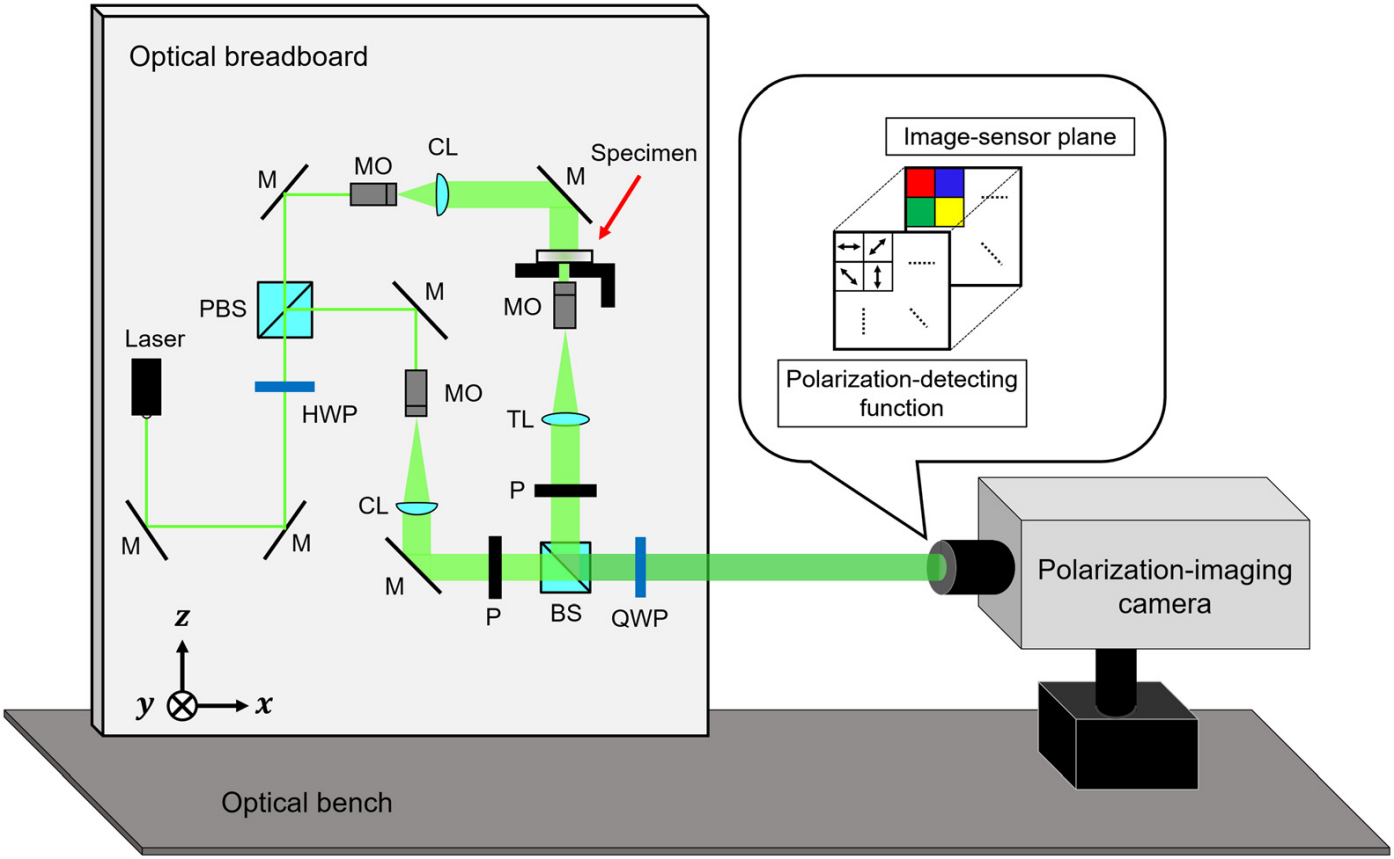
Schematic of the modularized microscope based on PPSDH. M, mirror; BS, beam splitter; PBS, polarizing beam splitter; HWP, half-wave plate; QWP, quarter-wave plate; P, polarizer; MO, microscope objective; TL, tube lens; CL, collimator lens.

Figure 3 shows a schematic of the optical system of the designed microscope. We defined the xyz coordinate system as shown in Fig. 3. The label of ⊗ denotes the direction of the y axis which points into the paper. The microscope consists of a laser, a Mach–Zehnder interferometer, a polarization-imaging camera, and a magnification optical system. The micropolarizer array is an array of 2×2 micropolarizers and functions as the phase-shifting array device. The micropolarizer array has a different polarization transmission axis for each 2×2  pixels and is equipped in front of the image sensor of the polarization-imaging camera. Hence, each 2×2  pixels of the polarization-imaging camera can record an interference fringe pattern containing four phase-shifted holograms. An afocal system was adopted as the magnification optical system. Since the lateral magnification of the magnification optical system is varied depending on the depth position in general, we need some numerical processes for resizing the reconstructed image to obtain the actual size of the object. By contrast, the lateral magnification of an afocal system is constant at arbitrary depth position. Therefore, an afocal system is useful for digital refocusing because the actual size of the object is easily obtained without resizing in digital refocusing. Then, a 4f-afocal system consisting of two convex lenses separated by the sum of their focal lengths was adopted for the magnification optical system of the microscope. In the afocal system, the lateral magnification ($M_{\mathrm{lat}}$) and the longitudinal magnification ($M_{\text{long}}$) can be written as

$$M_{\mathrm{lat}}=\frac{f_{\mathrm{mo}}}{f_{\mathrm{tl}}},$$ (1)

$$M_{\text{long}}={\left(M_{\mathrm{lat}}\right)}^{2}={\left(\frac{f_{\mathrm{mo}}}{f_{\mathrm{tl}}}\right)}^{2},$$ (2)

where $f_{\mathrm{mo}}$ and $f_{\mathrm{tl}}$ are the focal lengths of the microscope objective and that of the tube lens, respectively.

### 3.2 Construction

**Fig. 4:**
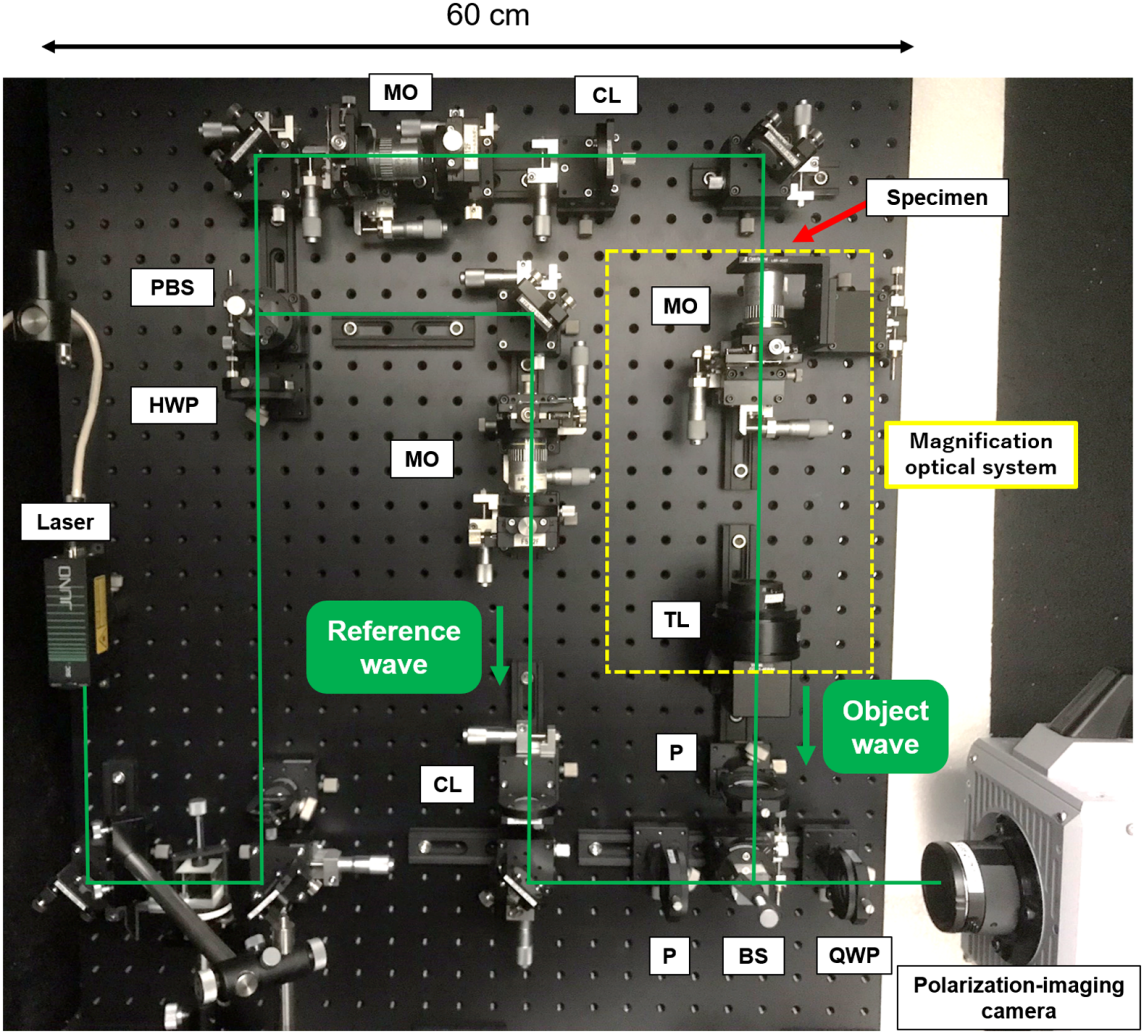
Photograph of the constructed optical system of the microscope. BS, beam splitter; PBS, polarizing beam splitter; HWP, half-wave plate; QWP, quarter-wave plate; P, polarizer; MO, microscope objective; TL, tube lens; CL, collimator lens. The green lines show the optical paths.

Figure 4 shows a photograph of the constructed optical system of the PPSDHM. The magnification optical system is shown in the yellow dashed line square in Fig. 4. The laser emits a linearly polarized light wave. After passing through a half-wave plate, the wave is split into an object illumination wave and a reference wave by a polarizing beam splitter. The reference wave is converted into a circularly polarized wave by a quarter-wave plate and is incident on the image sensor. Meanwhile, the object wave transmits the specimen and becomes the object wave. The object wave is converted into a circularly polarized wave by a quarter-wave plate and is incident on the image sensor. Each optical element was bolted on the optical breadboard. Each element in the breadboard was equipped with a two-axis translation stage for easily making fine and accurate adjustments for its position. The stage comes equipped with a metric micrometer for each axis. The height of the optical axis of the optical system with respect to the breadboard should be as low as possible to suppress the influence of external vibrations on each element. By considering the size of the optical elements, the lowest height from the breadboard surface was set to be 67 mm. It was required that the breadboard was not only large enough to integrate the optical elements, but also as small as possible to transport to another location. For this, we chose a breadboard of $600\times 900\;{\mathrm{mm}}^{2}$. An $\mathrm{Nd}:{\mathrm{YVO}}_{4}$ laser operated at 532 nm was used as a compact light source because the micropolarizer array of the polarization-imaging camera described below is optimized for this wavelength. In an afocal system, the higher the magnification is, the larger is the optical system. To achieve both high magnification and reduced path length on the breadboard, we adopted the system consisting of a microscope objective with a 20-mm focal length and a tube lens with a 200-mm focal length. Then, the lateral and the longitudinal magnifications are $M_{\mathrm{lat}}=10$ and $M_{\text{long}}=100$, respectively. A Photron FASTCAM-SA2-P, which can record a motion picture at the rate up to 86,400 frames per second (fps), was used as a polarization-imaging camera to record specimens dynamically moving in a micro-area. The pixel pitch of the polarization-imaging camera is 10  μm. The polarization-imaging camera was placed outside the breadboard because not only the size of the polarization-imaging camera is too large ($165\times 153\times 250\;{\mathrm{mm}}^{3}$), but also the weight is too heavy (6.9 kg) to stably fix on the breadboard. By doing so, the optical system has an advantage that the polarization-imaging camera can be easily replaced with a high-resolution one when slow-moving specimens are recorded. This is because there is a trade-off between speed and resolution in the high-speed camera. The total weight of the microscope is 33.3 kg, where most of the weight is consumed by the breadboard and the polarization-imaging camera, which accounts for 78.7% of the total weight. Considering the wavelength of the light, the numerical aperture of the microscope objective, and the ability of the polarization-imaging camera, the microscope provided the spatial resolution of 1.77  μm and the temporal resolution of 1/86,400 s.

## 4 Experiments and Results

### 4.1 Evaluation of Magnification

#### 4.1.1 Evaluation of lateral magnification

**Fig. 5:**
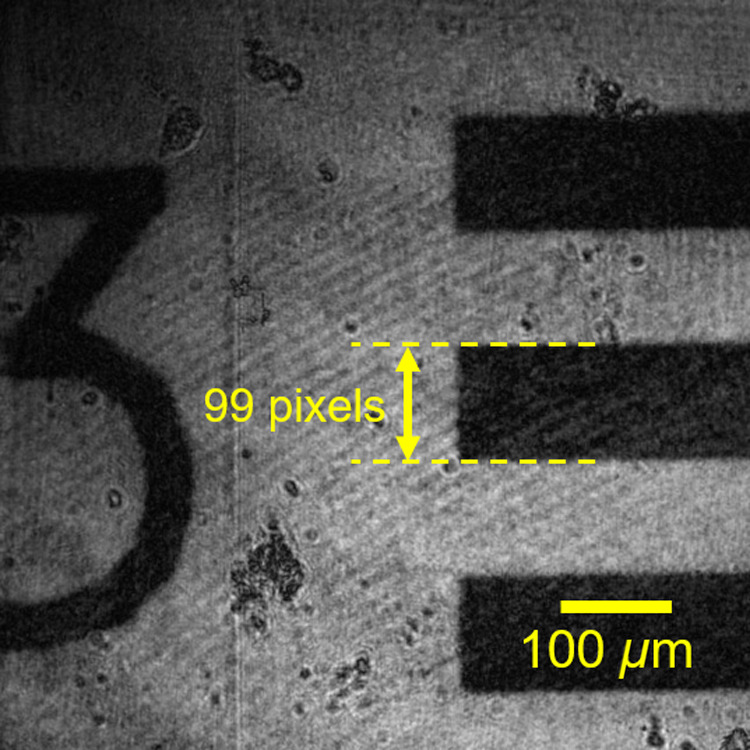
Reconstructed amplitude image for evaluating lateral magnification of the constructed microscope: the USAF test target was in focus.

First, the lateral magnification of the constructed PPSDHM was examined. A USAF test target was set as the specimen. A line with 99  μm (group 2, element 3 of the USAF test target) was recorded. Figure 5 shows the reconstructed amplitude image on which the USAF test target was in focus. The line width of group 2, element 3 in the image corresponded to 99 pixels; that is, 0.99 mm. Therefore, it was confirmed that the microscope provided 10× lateral magnification, which agreed with the design value.

#### 4.1.2 Evaluation of longitudinal magnification

**Fig. 6:**
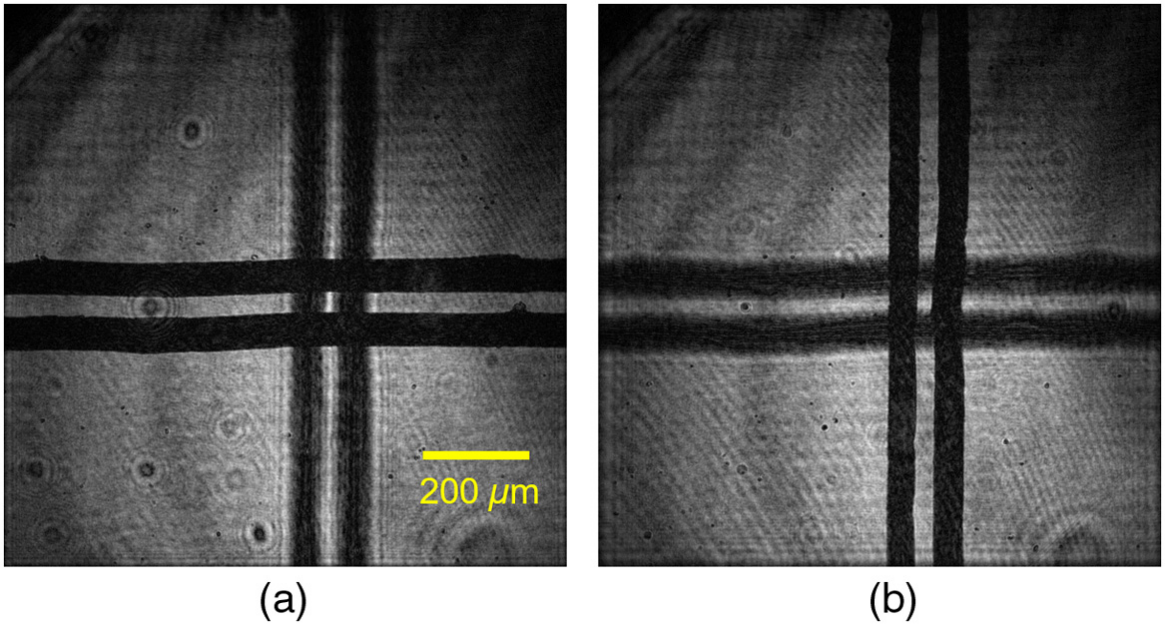
Reconstructed amplitude image at the experiment for evaluating longitudinal magnification of constructed microscope: (a) horizontal lines were in focus (z=25  mm) and (b) vertical lines were in focus (z=85  mm).

Next, the longitudinal magnification of the PPSDHM was examined. A slide glass of 0.91-mm thick was set for the specimen. A line pair was drawn on one side of the slide glass and another line pair intersecting the previous line pair was drawn on the other side. The longitudinal magnification of the microscope was examined by the difference of the depth position where each line pair was in focus. Figure 6 shows the reconstructed amplitude images at the depth position where each line pair was in focus. z means the distance away from the image sensor plane. The difference of the depth position was 60 mm. The obtained thickness of the slide glass was 91 mm considering the refractive index of the slide glass (n=1.52). Therefore, the microscope provided 100× longitudinal magnification, which agrees with the designed value.

Thus, we confirmed that the microscope gave the design magnification.

### 4.2 3-D Tracking of Moving Volvox

We conducted an experiment to demonstrate the effectiveness of the constructed microscope in terms of 3D measurements of living biospecimens. *Volvoxes* moving three-dimensionally in water were set as biospecimens.

#### 4.2.1 Experimental set up

**Fig. 7:**
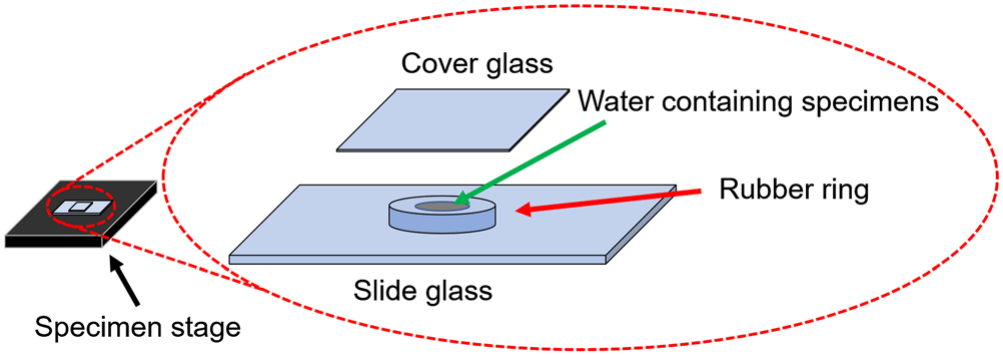
Schematic of the specimen setting.

Figure 7 shows a schematic of the specimen setting. A rubber ring was placed on a slide glass. The inside of the ring was filled with water containing specimens. To prevent the water surface from acting like a lens, the ring was covered with a cover glass. The number of the recording pixels, the recording frame rate, the shutter speed, and the total recording time were 1536×1536  pixels, 1000 fps, 0.25 ms, and 3.45 s, respectively. We chose 1000 fps only because this frame rate provided a large field of view suitable for the moving *Volvoxes*.

#### 4.2.2 Results

**Fig. 8:**
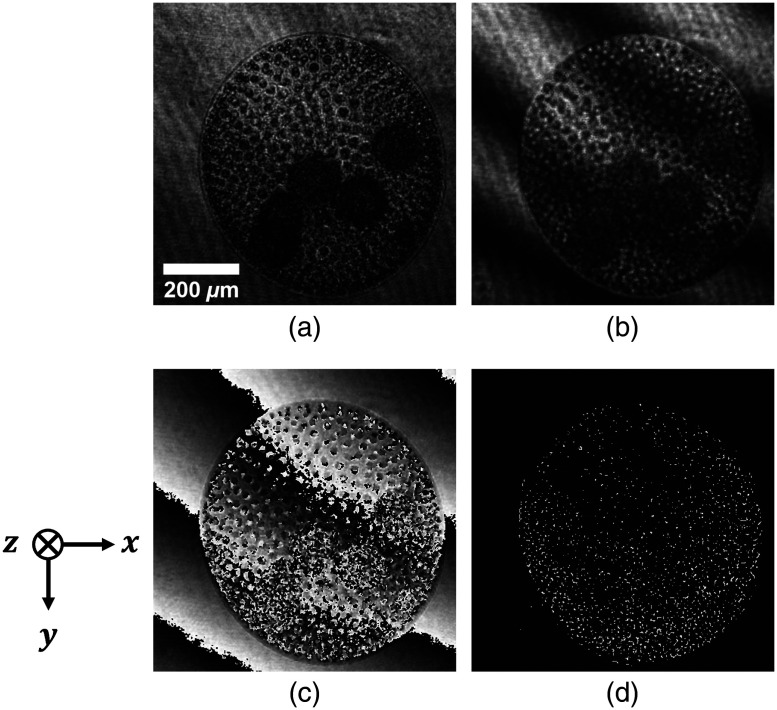
Reconstructed images from the recorded hologram: (a) amplitude image and (c) phase image reconstructed by the PPSDHM, (b) amplitude image and (d) phase image reconstructed by an in-line DHM without the phase shifting.

We reconstructed amplitude images and phase images from the recorded holograms. Figures 8(a) and 8(c) show the amplitude image and the phase image reconstructed by the PPSDHM. Figures 8(b) and 8(d) show the amplitude image and the phase image reconstructed by an in-line DHM without the phase-shifting for comparison. The scale bar shown in Fig. 8(a) was measured in the image space. In the images reconstructed by the in-line DHM without the phase-shifting, the edge of the *Volvox* appears blurred due to the superimposition of undesired images on the desired image. By contrast, the reconstructed images by the PPSDHM were free from any unwanted images. As seen from the results, it was confirmed that the PPSDHM was capable of obtaining the higher quality images.

**Fig. 9:**
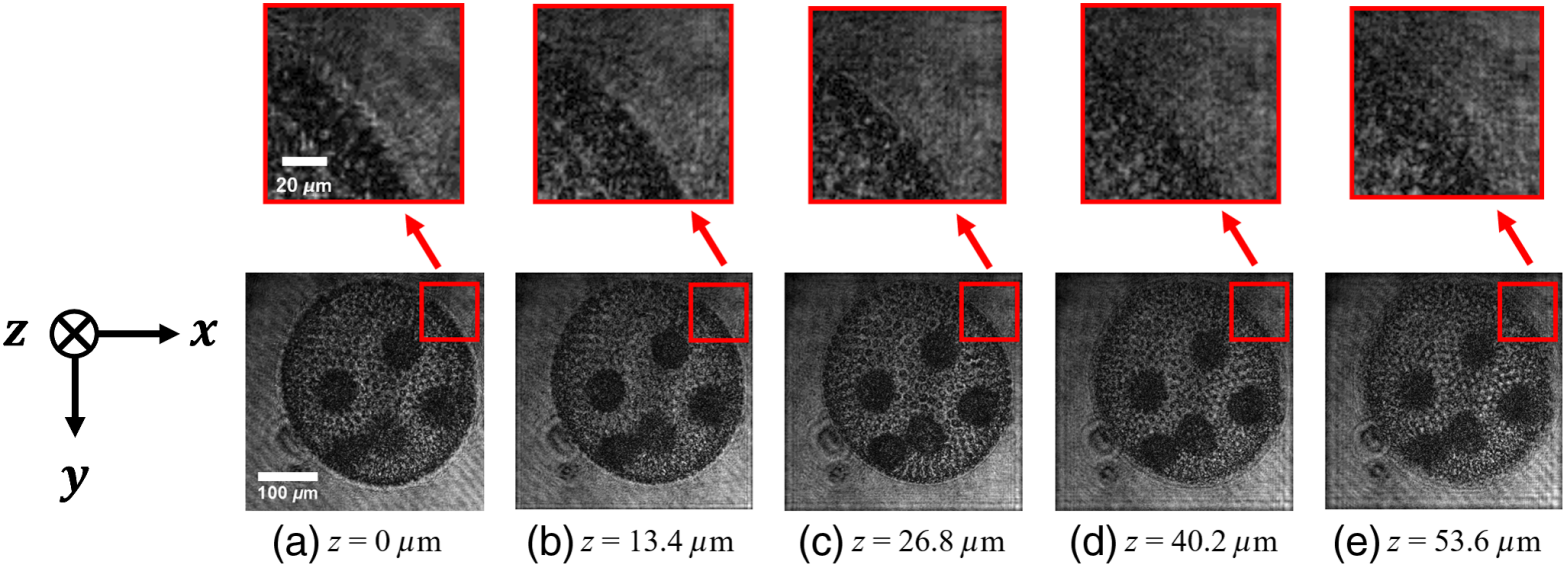
Amplitude images reconstructed from one of the holograms recorded by the constructed PPSDHM. The edge of the *Volvox* is in focus at the position 26.8  μm away from the image sensor.

Figure 9 shows the reconstructed amplitude images of the *Volvox* and the enlarged images of the edge of the *Volvox*. These images were obtained while the distance z was varied in the numerical processing for reconstruction of the hologram. The edge of the *Volvox* was the clearest at z=26.8  μm. By contrast, the edge of the *Volvox* was blurred at the other depth position. Thus, we succeeded in digital refocusing of the *Volvox* using the constructed PPSDHM.

**Fig. 10:**
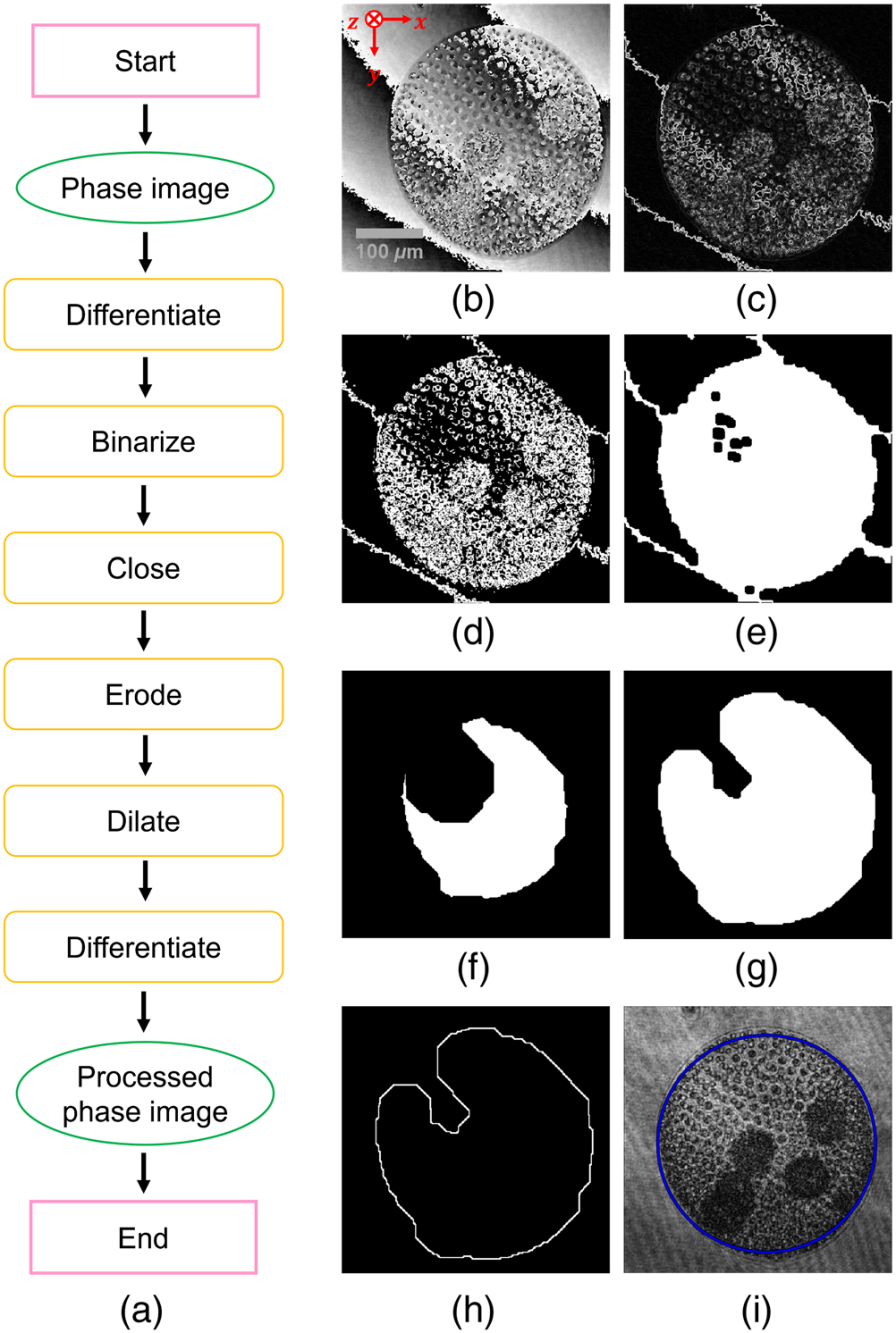
Processing flow for detecting the x and y coordinates of the center of the circle: (a) flowchart of the image processing, (b) reconstructed phase image, (c) previously differentiated image, (d) binarized image, (e) closed image, (f) eroded image, (g) dilated image, (h) later differentiated image, and (i) the blue circle representing the detected *Volvox*.

Next, to obtain the trajectories of the *Volvoxes*, each *Volvox* was assumed as a sphere. The trajectory of the *Volvox* was defined as that of the center of the sphere. To obtain x and y coordinates of the center, the *Volvox* was detected as a circle in the horizontal plane, which is parallel to the xy-plane, at the depth where the edge of the *Volvox* was in-focus. For this purpose, the following processes were applied to the reconstructed phase image according to the flowchart shown in Fig. 10(a).

(1) Differentiating the reconstructed phase image to enhance the contours of the *Volvox*.
(2) Binarizing the result of the process (1).
(3) Morphologically closing the result of the process (2); The morphological close operation is a dilation followed by an erosion. By this operation, each value of the pixels surrounded by the edge of the *Volvox* is changed to 255.
(4) Eroding the result of the process (3) to remove the fringe in the background.
(5) Dilating the result of the process (4) to restore the size of the *Volvox* image to the original size.
(6) Differentiating the result of the process (5) to extract the contours of the *Volvox*.

The circle representing the perimeter of the *Volvox* was detected in the processed image. Figures 10(b)–10(i) show an example set of the processed images and the detected circle. The lateral position of the center of the *Volvox* was determined as the x and y coordinates of the center of the circle. As shown in Fig. 8, the depth positions were determined as the z coordinate where the edge of the *Volvox* in the reconstructed amplitude image was the clearest.

**Fig. 11:**
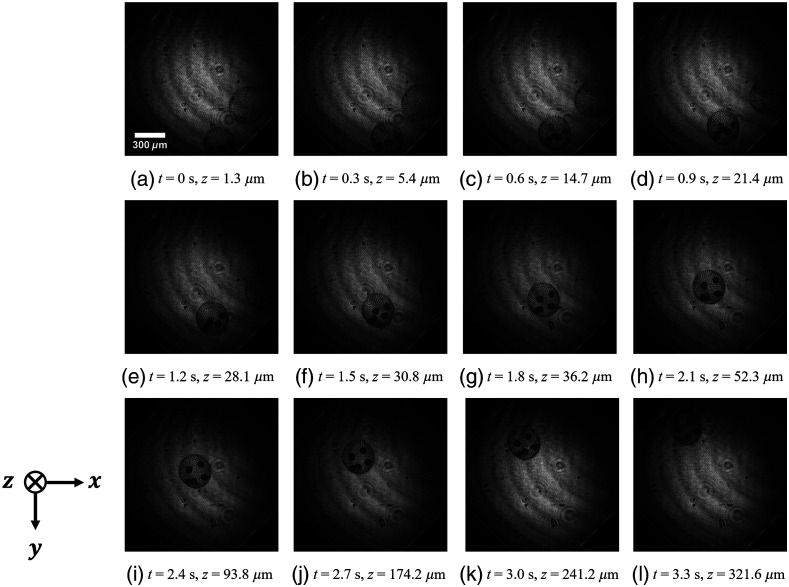
Reconstructed amplitude images extracted from every 300 frames: (a)–(l) the edge of the *Volvox*A was in focus at each frame.

Figure 11 shows the reconstructed amplitude images. The images were the in-focus images of the edge of the *Volvox* when the *Volvox* was moving. The time interval between the adjacent image corresponds to 0.3 s. At the start of recording (t=0  s), we name the *Volvox* in the smaller x coordinate and that in the larger x coordinate as *Volvox*A and *Volvox*B, respectively. *Volvox*A moved in the negative directions of the x and y axes and away from the image sensor in the depth direction. Meanwhile, *Volvox*B moved in the positive direction of the x axis and the negative direction of the y axis and away from the image sensor in the depth direction.

**Fig. 12:**
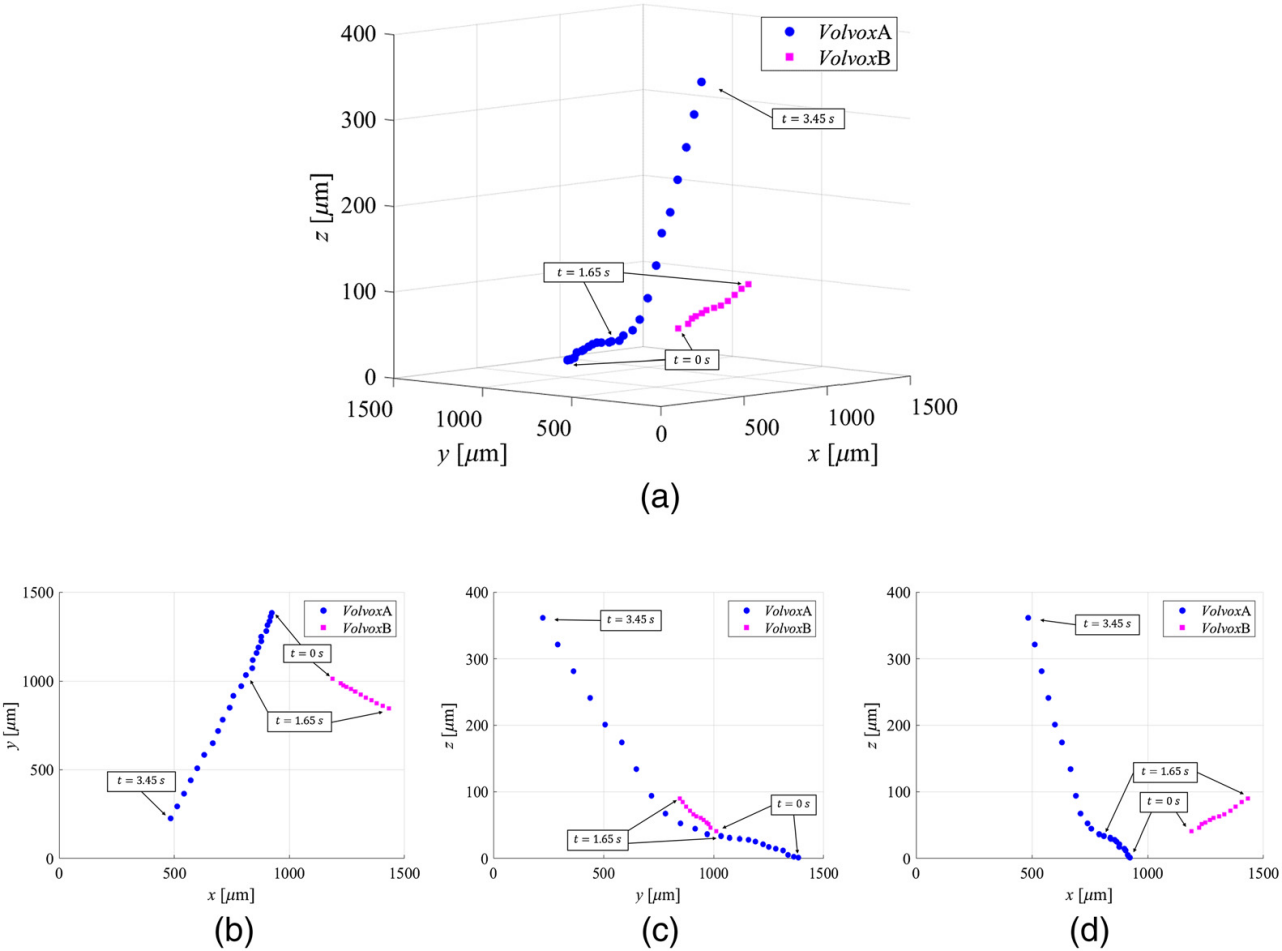
Trajectories of the centers of the *Volvoxes* moving in water plotted every 150 frames: Trajectories in (a) 3D, (b) the xy-plane, (c) the yz-plane, and (b) the xz-plane.

Figure 12 shows the 3D trajectories of the centers of the *Volvox*A and *Volvox*B moving in water. The address of the uppermost and leftmost pixel was defined as (x,y)=(1,1) in each reconstructed image. *Volvox*A moved slowly (at 223.5  μm/s in the lateral direction and 19.4  μm/s in the depth direction) until t=1.65  s, but then the moving speed increased to 489.2  μm/s in the lateral direction and 197.3  μm/s in the depth direction to reach the edge of the imaging area. *Volvox*A moved 1.29 mm in the 3D area at 374.6  μm/s through the recording time. By contrast, the x coordinate of the center of *Volvox*B was 1188.4  μm at t=0, and *Volvox*B moved in the positive direction of the x axis and out of the imaging area. *Volvox*B moved 0.3 mm in the 3D area at 181.8  μm/s (at 179.0  μm/s in the lateral direction and 30.0  μm/s in the depth direction) for 1.65 s. The speed of *Volvox*B was less than half that of *Volvox*A. Thus, we succeeded in the 3D tracking of moving *Volvoxes*.

## 5 Discussion

We describe the factors that limit the reduction in size of the constructed PPSDHM. In this PPSDHM, the alignment can be adjusted. Furthermore, the light intensity ratio between the object wave and the reference wave depending on the objects to appropriately record holograms can be adjusted in this PPSDHM. For this reason, this PPSDHM was designed as a two-beam interference optical system, which became larger and more complex. Furthermore, the polarization state had to be precisely adjusted in our system because the parallel phase shifting in this system was implemented by making good use of the polarization. Thus, this PPSDHM required the additional space for arranging the optical elements to adjust the polarization states of the object wave and the reference wave. In addition, the afocal system was adopted in this PPSDHM for magnification. Therefore, the length of the optical path of the object wave in our system needs to include the focal length of the microscope objective and that of the tube lens, thus making our system larger. Moreover, since the optical system was custom built by us using off the shelf components, it occupies a larger size. The above factors put a limit on making this PPSDHM system any smaller. It will be possible to pack this PPSDHM more compactly in the future by constructing the optical system with high precision in advance so that each adjustment part becomes unnecessary.

Next, we describe the determination method of the depth position of the object in each experiment. We manually determined the depth positions of the objects from the reconstructed amplitude images. The automatic depth measurement from a digital hologram is important on 3D measurements because it saves a lot of time and man hours and realizes more accurate determination.^37^^,^^38^ Therefore, we are considering automatic depth measurement for this PPSDHM in the future.

Finally, we describe the result of the 3D tracking of *Volvox*. In a DHM, the aberrations caused in the object wave and the reference wave are usually corrected. Thus, we attempted to correct the aberrations in Fig. 8(c), but we carried out the experiment without correcting the aberrations because it became difficult to obtain the lateral position of the *Volvox* by the effect of the internal refractive index of the *Volvox*. The aberrations are estimated to be about 1  μm in the depth direction from the reconstructed phase image. The error by the aberrations is negligibly small for tracking the *Volvox* moving more than 300  μm in the depth direction. Therefore, we conclude that the trajectories shown in Fig. 12 are sufficient enough to understand the 3D behavior of the *Volvoxes*.

## 6 Conclusion

We designed and constructed a modularized PPSDHM which is capable of 3D image acquisition. The optical elements of the microscope were integrated on the optical breadboard. The constructed microscope can record transparent objects and be transported to another location. In order to demonstrate the effectiveness of the microscope, we recorded *Volvoxes* moving in 3D as dynamic specimens. The image of the object, which was free from the undesired images, was successfully reconstructed from the recorded hologram. The trajectories of the *Volvoxes* moving in a micro-area were obtained from the reconstructed images. The recording capability of high-speed motion-picture images of dynamic specimens was experimentally demonstrated by the microscope. This system will contribute to reveal the 3D behaviors of various cells and microorganisms that have not been clarified.
